# Zeolite Heulandite Modified with N,N′-bis(3-Triethoxysilylpropyl)thiourea—Adsorption of Ni(II) and Cu(II) Ions: A Quantum Chemical Insight into the Mechanism

**DOI:** 10.3390/molecules30244811

**Published:** 2025-12-17

**Authors:** Elena G. Filatova, Arailym M. Nalibayeva, Oksana V. Lebedeva, Sergey A. Beznosyuk, Andrey V. Ryabykh, Elizaveta N. Oborina, Yerlan N. Abdikalykov, Mirgul Zh. Turmukhanova, Igor B. Rozentsveig, Sergey N. Adamovich

**Affiliations:** 1 Department of Chemistry and Biotechnology Named After V.V. Tuturina, Irkutsk National Research Technical University, 83 Lermontov Street, 664074 Irkutsk, Russia; efila@list.ru (E.G.F.);; 2 D.V. Sokolskiy Institute of Fuel, Catalysis and Electrochemistry, 142 Kunaev Street, 050010 Almaty, Kazakhstan; a.nalibayeva@ifce.kz (A.M.N.); y.abdikalykov@ifce.kz (Y.N.A.); 3 Department of Physical and Inorganic Chemistry, Altai State University, 61 Lenin Avenue, 656049 Barnaul, Russia; beznosyuk@chem.asu.ru (S.A.B.);; 4 A.E. Favorsky Irkutsk Institute of Chemistry, Siberian Branch of the Russian Academy of Sciences, 1 Favorsky Street, 664033 Irkutsk, Russia; 5 Faculty of Chemistry and Chemical Technology, Al-Farabi Kazakh National University, 71 Al-Farabi Avenue, 050040 Almaty, Kazakhstan; t_mirgul@mail.ru

**Keywords:** natural zeolite, N,N′-bis(3-triethoxysilylpropyl)thiourea, modification, adsorption, heavy metals, quantum chemical modeling

## Abstract

A new sorption material (GS) was obtained by the modification of heulandite zeolite (G) with N,N′-bis-(3-triethoxysilylpropyl)thiocarbamide (S). The composition, structure, and surface morphology of the GS material were confirmed using elemental analysis, IR-, NMR-spectroscopy, X-ray diffraction, scanning electron microscopy (SEM), energy dispersive X-ray spectroscopy (EDX), elemental mapping, and nitrogen adsorption/desorption (BET). The potential of GS as a sorbent for the removal of Cu(II) and Ni(II) ions from concentrated solutions was demonstrated. The nature of the adsorption of Cu(II) and Ni(II) ions was investigated using the Langmuir, Freundlich, and Dubinin–Radushkevich models. The adsorption value of Cu(II) and Ni(II) ions by the GS sorbent was found to be 1.7 and 2.1 times higher than that of heulandite, amounting to 0.128 mmol/g (8.1 mg/g) and 0.214 mmol/g (12.6 mg/g), respectively. The free energy of adsorption E for the adsorption of Cu(II) and Ni(II) ions was determined to be 12.5 and 16.2 kJ/mol, respectively. Calculations of changes in Gibbs energy based on quantum chemical modeling results ($\Delta G_{298}^{0}$ = −38.5 kJ/mol for Ni and $\Delta G_{298}^{0}$ = −56.5 kJ/mol for Cu) confirmed that adsorption of heavy metal ions onto the GS sample occurs through the formation of metal ion coordination complexes with the sorbent’s functional groups (chemosorption). The proposed method of obtaining new sorption materials based on natural heulandite is straightforward and cost-effective, enabling the production of high-capacity sorption products.

## 1. Introduction

Today, heavy metal ions (HMIs) are considered priority pollutants that must be monitored in all environments [1,2,3]. Unlike other organic and inorganic pollutants, HMIs are not subjected to biological degradation, but they are capable of accumulating in living organisms, including plants, animals, and humans, with increasing concentrations as they move up the food chain. ITMs are known to form complexes with sulfur-, oxygen-, or nitrogen-containing compounds and to inactivate enzyme systems and protein structures, leading to cellular dysfunction [4,5]. Sorption methods remain unrivaled for removing HMIs from aqueous solutions [6,7,8], as they allow for the most complete removal of contaminants. When selecting sorbents, natural zeolites are often preferred [9,10,11,12]. Natural zeolites attract a lot of attention due to their excellent sorption properties and their ability to remove cations and small amounts of heavy metal ions from aqueous solutions via ion exchange [13]. To improve their sorption properties, natural zeolites are modified with mineral [14,15,16,17,18,19] and organic [20,21,22,23] compounds.

Silylation is a promising method for modifying zeolite, ensuring the stable immobilization of functional organic groups on the surface [24,25,26,27]. This process involves a reaction between -SiCl_3_ or -Si(OAlk)_3_ groups and OH moiety of the zeolite to form a hybrid organo–inorganic layer. Among functional compounds, thiosemicarbazide derivatives are widely used [28,29,30]. The thiosemicarbazide group is effective in sorption processes due to its good ability to form complexes with metals [31,32,33].

Organosilicon thiourea derivatives, such as N′-bis(3-triethoxysilylpropyl)thiocarbamide, can also be used to modify zeolites [34,35,36]. The presence of a thiocarbamide group, -HN-C(S)-NH-, enhances the adsorption capacity of zeolites compared to natural minerals.

The adsorption mechanism on natural zeolites can be either an ion-exchange process or a physical process [37,38,39], with adsorption isotherms being well described by the Langmuir and Freundlich models [40,41,42]. The kinetics of ion-exchange adsorption are best described by second-order models [43,44,45].

Some of the most common natural zeolites are clinoptilolite and heulandite (Table 1), which are recognized as effective sorbents for removing HMIs, including copper (II) and nickel (II) ions.

The Si/Al ratio determines the hydrophobicity, acidity, and stability of zeolites. Those with a high Si/Al ratio are more hydrophobic and thermally stable, while zeolites with a low Si/Al ratio have a greater cation exchange capacity and stronger interaction with polar molecules [46].

For the first time in this work, heulandite (zeolite with a high capacity for cation exchange) was used as a carrier for the application of a new modifier, N,N′-bis-(3-triethoxysilylpropyl)thiocarbamide.

The proposed method of obtaining new sorption materials involves a single-stage process carried out at a low temperature and atmospheric pressure. This method enables the production of modern composites with high sorption capacity. Such sorbents are expected to demonstrate enhanced HMI sorption intensity and are recommended for the extraction of copper and nickel from highly concentrated and toxic-spent process solutions and industrial wastewater.

The objective of this study was to provide a comprehensive understanding of the mechanism of Ni(II) and Cu(II) ion adsorption on a heulandite cluster modified with N,N′-bis(3-triethoxy-silylpropyl)thiocarbamide, as substantiated by quantum chemical calculations.

## 2. Results and Discussion

### 2.1. Composition and Structure of GS

The ion exchange properties of heulandite in relation to divalent Cu(II) and Ni(II) ions were previously studied [47]. However, modifying heulandite with nitrogen- and sulfur-containing organosilicon compounds makes it possible to obtain sorption materials with improved functional properties.

Heulandite (**G**) was modified with N,N′-bis(3-triethoxysilylpropyl)thiocarbamide (**S**). Due to the presence of chemically active thiourea groups in the finish, adsorption in this case may be accompanied by the formation of stable coordination compounds on the surface of the material, determining the materials’ high efficiency. According to elemental analysis methods (nitrogen content: 5.3%, sulfur content: 4.8%), the content of the finish in the obtained modified products is 9.4%.

**Scheme 1:**
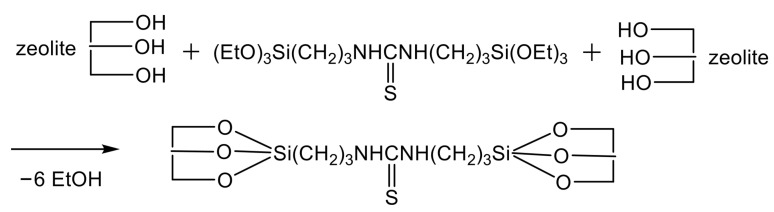
Immobilization of N,N′-bis(3-triethoxysilylpropyl)thiourea.

Immobilization of symmetric N,N′-bis(3-triethoxysilylpropyl)thiocarbamide (**S**), having two terminal groups Si(OEt)_3_ on the surface of (**G**), is accompanied by its interaction with the surface OH groups of the mineral to form an “arched” structure (Scheme 1).

The high degree of affinity between the natural carrier and the synthetic finish ensures the stability of the modified sorbent, largely determined by the formation of multiple Si-O-Si bonds during the aluminosilicate immobilization. The modified sample does remain unchanged in mass upon contact with concentrated solutions of mineral acids (HCl, H_2_SO_4_ at 5 mol/dm^3^) and organic solvents (ethyl alcohol, dioxane, tetrahydrofuran, etc.).

**Figure 1:**
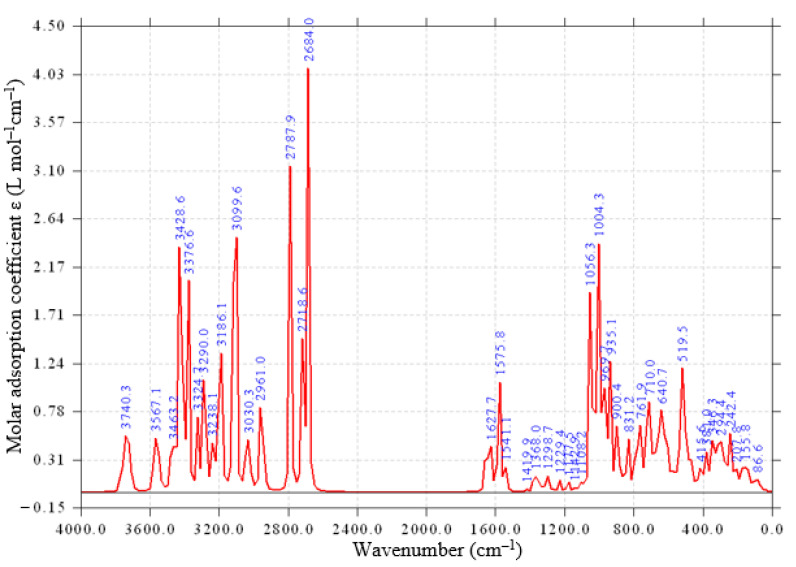
IR spectrum of **GS**.

The structure of modified heulandite **GS** is confirmed by the presence of bands of N–H bond valence vibrations (3290, 2961, 1627, and 1575 cm^−1^), set of thiocarbonyl C=S group bands (1419–1229 cm^−1^), and intense band of Si-O bond valence vibrations (1056 cm^−1^) in its IR spectrum (Figure 1).

The presence of thiocarbamide groups in the organosilicon finish enables the formation of Cu(II) and Ni(II) ion-coordination complexes with donor nitrogen and sulfur atoms on the surface of the immobilized sorbent.

**Figure 2:**
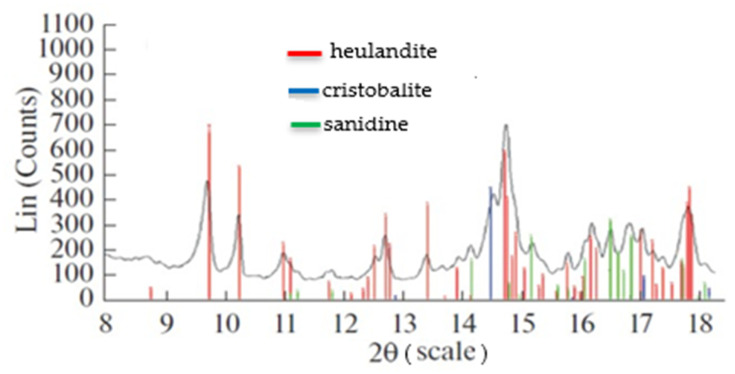
Diffraction pattern of zeolite modified with **S**.

X-ray diffraction analysis demonstrated that **S**-modified aluminosilicates comprise the following components: heulandite Ca[Al_2_Si_7_O_18_]·6H_2_O, sanidine KAlSi_3_O_8_, and cristobalite SiO_2_ (Figure 2). During the modification process, the content of heulandite decreases due to the introduction of N,N′-bis(3-triethoxysilylpropyl)thiocarbamide into the sorbent composition.

The textural characteristics of the obtained sorbents were determined using the low-temperature adsorption-desorption method. The results revealed a specific surface area of 6.68 m^2^/g, a specific pore volume of 0.003 cm^3^/g, a microporous volume of 0.001 cm^3^/g, and an average pore size of 1.82 nm.

### 2.2. Adsorption Properties of GS

The **GS** sample exhibits adsorption activity with respect to Cu(II) and Ni(II) ions. The adsorption of these ions by the **GS** sorbent was studied by constructing and analyzing adsorption isotherms. The method of constant weights and variable concentrations was employed for this purpose. The concentrations of the heavy metal ions in the solution were 5, 10, 20, 40, 60, 80, and 100 mg/dm^3^; the mass of the sorbent was 1 g; and the volume of the solution was 0.1 dm^3^. The temperature was 298 K, and the pH was 5–5.5 for Ni(II) and Cu(II). Complete adsorption equilibrium was achieved after 2 h of contact between the sorbent and the adsorbate solution.

**Scheme 2:**
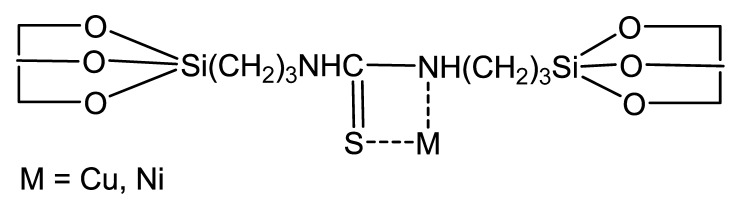
Formation of coordination compounds on the **GS** surface.

It is assumed that the extraction of HMIs by the studied sorbent **GS** is the result of chemisorption, which is caused by the formation of coordination complexes with Cu(II) and Ni(II) ions [48]. This explains the high efficiency of such materials (Scheme 2).

**Figure 3:**
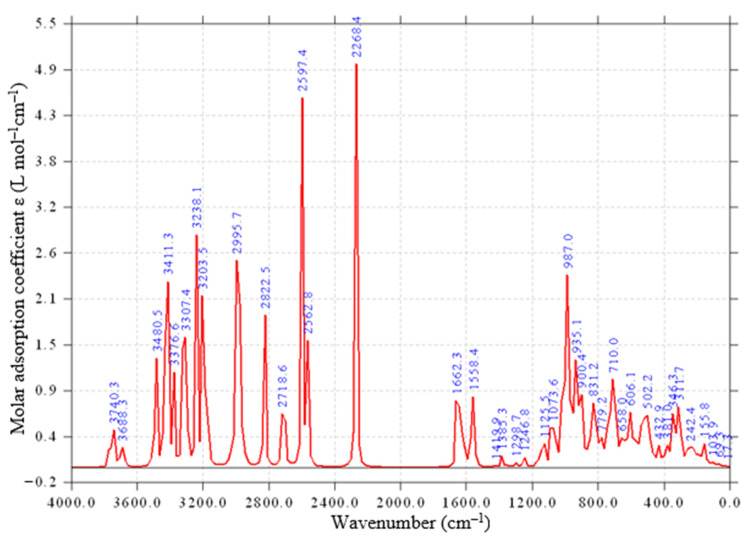
IR spectrum of GS-Cu.

In the IR spectrum of the **GS** sorbent after adsorption of Cu(II) ions (Figure 3), the bands of valence vibrations of the N–H bond shift by 17 cm^−1^ (to 3307 cm^−1^ and to 1568 cm^−1^) and by 35 cm^−1^ (to 1662 cm^−1^). In addition, the picture changes noticeably in the region of 1419–1229 cm^−1^ (C=S). These changes confirm the possibility of bond formation between the Cu(II) ion and the nitrogen and sulfur atoms of the thiourea group **GS** via a donor-acceptor mechanism.

**Figure 4:**
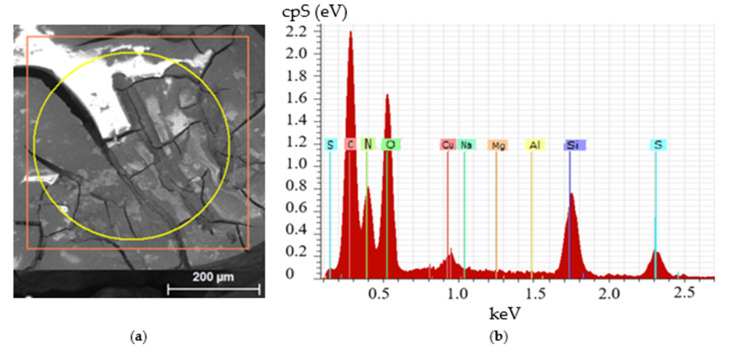
**GS-Cu** sample: (**a**) SEM image and (**b**) EDX spectra.

The SEM image, EDX, and elemental mapping results for the **GS** sample after copper adsorption are shown in Figure 4.

**Figure 5:**
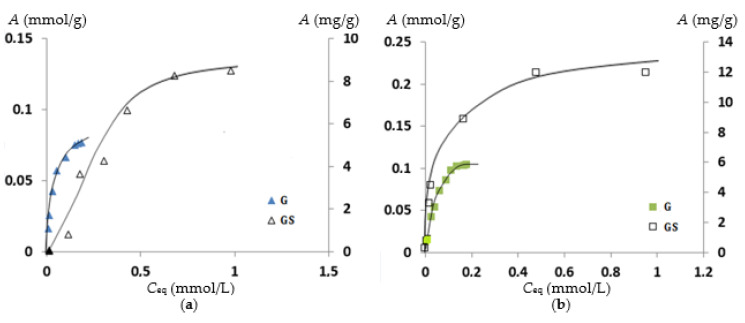
Adsorption isotherms **G** and **GS** of Cu(II) (**a**) and Ni(II) (**b**) ions.

No noticeable structural changes occurred on the surface of the sorbents under study as a result of adsorption; the Cu(II) ion content in the modified sample was at least 1.95 wt.%. These results are consistent with the adsorption capacities determined from the analysis of adsorption isotherms for Cu(II) (**a**) and Ni(II) (**b**) (Figure 5).

The adsorption values of Cu(II) and Ni(II) ions by the **GS** sorbent were 1.7 and 2.1 times higher than those of natural heulandite **G**, amounting to 0.128 mmol/g (8.1 mg/g) and 0.214 mmol/g (12.6 mg/g), respectively.

### 2.3. Models of Adsorption

**Figure 6:**
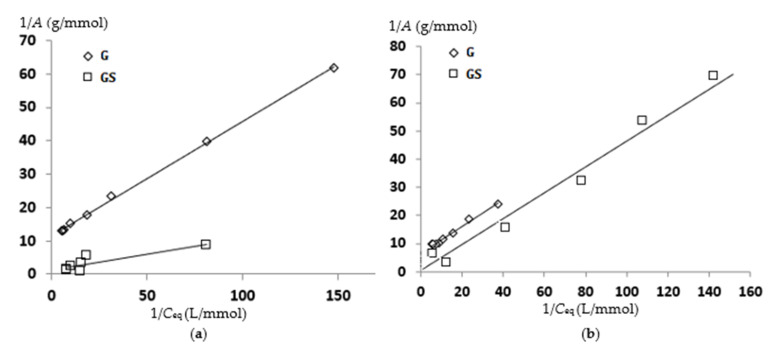
Langmuir adsorption isotherms **G** and **GS** of Cu(II) (**a**) and Ni(II) ions (**b**).

The Langmuir, Freundlich, and Dubinin–Radushkevich models [31,40,41,42] were used to describe the adsorption. The type of isotherms obtained is closest to Langmuir adsorption isotherms. Figure 6 shows the linear forms of Langmuir isotherms of **G** and **GS** of Cu(II) (**a**) and Ni(II) (**b**) ions.

The characteristics of the Langmuir isotherm of **G** and **GS** can be expressed by a dimensionless constant known as the separation coefficient (or equilibrium parameter) *R_L_*. Adsorption is considered irreversible when *R_L_* = 0, favorable when 0 < *R_L_* <1, linear when *R_L_* = 1, and unfavorable when *R_L_* > 1 [49]. The maximum adsorption value, *A*_∞_, was determined by the length of the line segment intersecting the *y*-axis. The adsorption equilibrium constant *K* was determined by the tangent to the slope of the line. The values are given in Table 2.

The values of the separation coefficient *R_L_* are in the range 0–1 (Table 2). This indicates favorable adsorption of HMIs by the sorbents under study. Analysis of the determination coefficients 0.999 shows that the Langmuir model best describes the adsorption of Cu(II) ions for sample **G.** In the case of **G** and **GS**, ion exchange and chemisorption presumably take place [11,34]. The maximum adsorption value of Ni(II) ions is 0.781 mmol/g (46.1 mg/g) for the **GS** sorbent, which is 5.2 times higher than that of natural aluminosilicates **G**. The maximum adsorption value for Cu(II) ions is 0.468 mmol/g (29.7 mg/g), which is 5.4 times higher than that of **G**.

**Figure 7:**
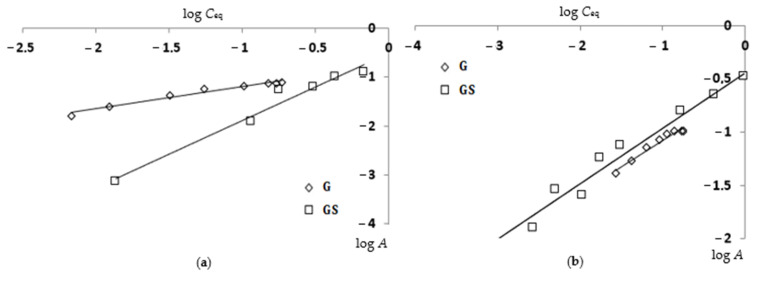
Freundlich adsorption isotherms of Cu(II) (**a**) and Ni(II) ions (**b**).

Freundlich’s empirical equation is widely used in the field of medium fillings of a heterogeneous sorbent surface [49,50]. According to Freundlich’s model, adsorption centers have different energy values, and active adsorption centers with maximum energy are filled first. The constants *K_f_* and *n* enable the adsorption capacity of modified minerals to be compared. At an HMI concentration of 1 mol/dm^3^, the adsorption value of these ions will be equal to *K_f_*. Consequently, *K_f_* is often referred to as the molar adsorption coefficient. For the HMIs under study, linear dependencies in logarithmic form were constructed for the Freundlich equation (Figure 7).

The constants *n* and *K_f_* (Table 3) were determined based on the slopes of the lines shown in Figure 7 and the segment cut off by the ordinate axis. The molar coefficient reaches its maximum value during the adsorption of Ni(II) ions by the **G** and **GS** sorbents. The **GS** sample exhibited the highest adsorption values for Cu(II) and Ni(II) ions, at 0.307 and 0.358 mol/dm^3^, respectively. The parameter *n* indicates the intensity of the sorbent–adsorbate interaction. The strongest interaction was found between HMIs samples **G** and **GS**.

**Figure 8:**
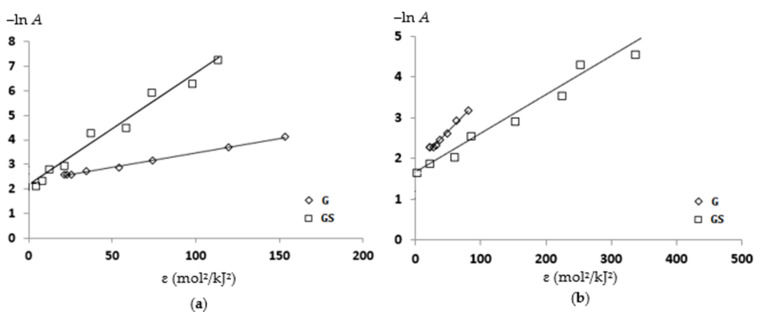
Dubinin–Radushkevich adsorption isotherms of Cu(II) (**a**) and Ni(II) ions (**b**).

The Dubinin–Radushkevich adsorption model was also employed to describe the experimental data. Based on the Dubinin–Radushkevich equation in logarithmic form, the slope of the lines (Figure 8) and the segment cut off by the ordinate axis were used to determine the constants *k* and *A_m_* (Table 4).

The Dubinin–Radushkevich model also indicates the nature of adsorption of the adsorbate onto the sorbent and can be used to calculate the free energy of adsorption *E* = (−2*k*) ^−0.5^. The results obtained and the values of *E* are presented in Table 5. It is known that if the value of the free energy of adsorption lies between 8 and 16 kJ/mol, then the adsorption process proceeds via chemosorption mechanism [51].

The free energy of adsorption for the **GS** sorbent during the adsorption of divalent HMIs ranges from 12.5 to 16.2 kJ/mol (Table 4). These data are confirmed by quantum chemical calculations, which show that the adsorption process can occur via a donor-acceptor interaction mechanism. In the case of natural zeolites, ion exchange proceeds with Ni(II) ions (*E* = 8.5 kJ/mol), while physical adsorption takes place with Cu(II) (*E* = 7.3 kJ/mol).

Analysis of the coefficients of determination for the Langmuir, Freundlich, and Dubinin–Radushkevich models indicates that these models all adequately describe the adsorption of HMIs onto aluminosilicates **GS** and **G**.

### 2.4. Quantum Chemical Study of the Adsorption Mechanism of GS by Heavy Metal Ions

A quantum chemical approach was used to elucidate the structural features of the **GS** adsorbent, which explain the increase in sorption capacity compared to heulandite **G**.

In heulandite **G**, calcium ions are strongly bound to oxygen atoms in the aluminosilicate framework via electrostatic, closed-shell interactions (ionic bond).

**Figure 9:**
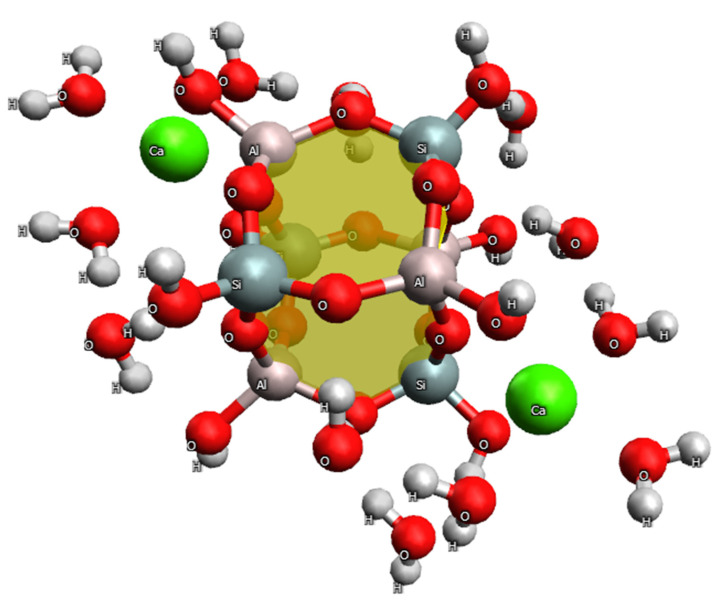
Heulandite cluster (G) with two calcium ions Ca(II) and twelve water molecules H_2_O.

Figure 9 shows the atomic–molecular structure of a heulandite cluster (**G**) with two calcium ions Ca(II) and twelve water molecules. Due to the presence of four aluminum atoms in a tetrahedral coordination environment (with a total of four additional hydroxide ions, OH^−^), the entire cluster structure has a total charge of −4, which is balanced by two calcium ions Ca(II).

All ions modeled separately from the cluster (either before adsorption, such as Ni(II) and Cu(II), or after adsorption, such as Ca(II)) are modeled with 12 H_2_O molecules.

**Figure 10:**
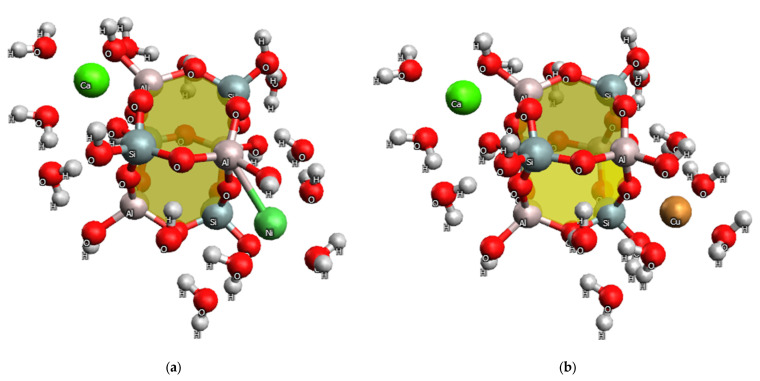
Heulandite cluster (**G**) with one calcium ion Ca(II), twelve water molecules H_2_O, and one Ni(II) ion (**a**) and one Cu(II) ion (**b**).

Modeling has shown that Ni(II) and Cu(II) ions add to the site of the detached Ca(II) ion (Figure 10).

The thermodynamics of ion adsorption on this cluster are presented in Table 5.

The negative value of the change in the Gibbs function indicates a thermodynamic advantage to the attachment of these ions. Furthermore, the Ni(II) ion is adsorbed with a greater release of energy than the Cu(II) ion. The absolute value of the change in the Gibbs function for the Ni(II) ion evidences the ion-exchange nature of adsorption, whereas for the Cu(II) ion, it indicates the physical nature of adsorption. As mentioned above, heulandite is a microporous sorbent. In the case of copper adsorption, the process occurs due to Van der Waals forces. According to the chemical reaction isotherm equation, the small value of $\Delta G_{298}^{0}$ also enables us to conclude that at high concentrations of Ni(II) or Cu(II) ions in the initial solution, the equilibrium may shift toward adsorption. This confirms the experimental data on the low adsorption of these ions onto heulandite **G**, with Ni(II) ions having a greater adsorption capacity.

**Figure 11:**
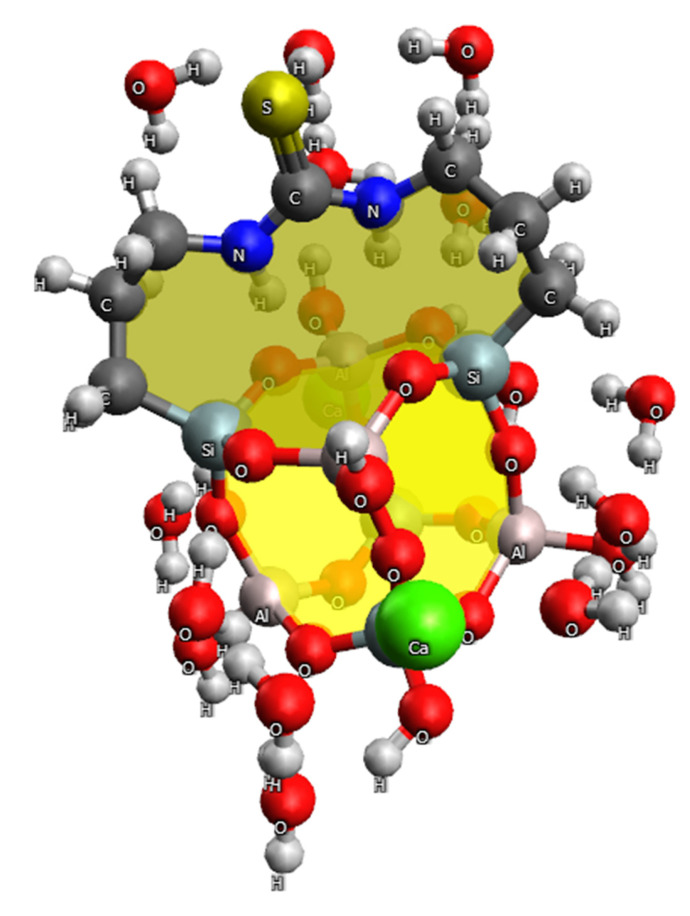
**GS** cluster with two calcium ions Ca(II) and twelve water molecules H_2_O.

Modification of the aluminosilicate structure with N,N′-bis-(3-triethoxysilylpropyl)thiocarbamide **S** weakens the bond between the oxygen and calcium atoms. This increases the desorption capacity of calcium ions, promoting the subsequent adsorption of Cu(II) and Ni(II) ions (Figure 11).

Three cases of thermodynamics of HMIs adsorption on the **GS** cluster were considered.

1. Thermodynamics of HMIs adsorption on the **GS** cluster without the desorption of calcium ions Ca(II).
2. Thermodynamics of HMIs adsorption on the **GS** cluster with the desorption of calcium ions Ca(II).
3. Thermodynamics of HMIs adsorption on the GS cluster with parallel desorption of calcium ions Ca(II) (ion exchange process).

**Figure 12:**
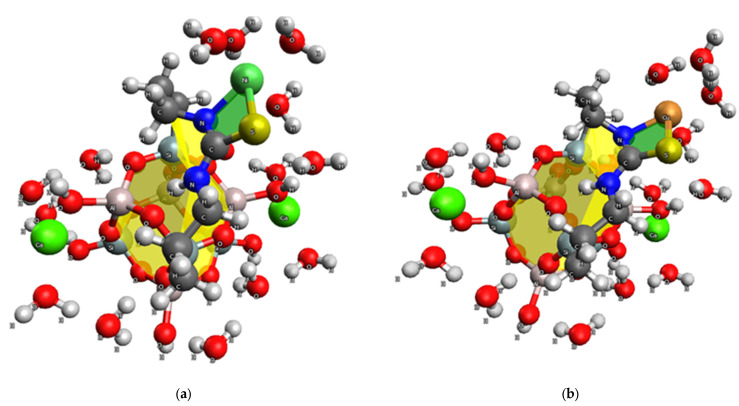
**GS** cluster with two Ca(II) ions, twelve H_2_O molecules, and one Ni(II) ion (**a**) and one Cu(II) ion (**b**).

The first case of modeling the adsorption of Ni(II) and Cu(II) ions onto the **GS** cluster without the desorption of Ca(II) ions was performed. This results in the formation of structures, as shown in Figure 12.

The thermodynamic functions for the addition of HMIs to **GS** without the desorption of Ca(II) ions were calculated (Table 6).

Large positive values of the Gibbs function changes indicate that it is unfavorable for Ni(II) and Cu(II) positive ions to add to a neutral cluster to form a positively charged cluster complex. At the same time, a stable minimum is observed when Ni(II) and Cu(II) ions form bonds with nitrogen atoms (**S**).

Evaluating the thermodynamics of Ca(II) ion desorption based on the Gibbs function change values indicates that Ca(II) ions spontaneously desorb in an aqueous medium when heulandite **G** is modified with **S** (Table 7). This was not observed in heulandite **G** ($\Delta G_{298}^{0}$ = +37.96). Consequently, the cluster becomes charged with a charge of -2, promoting the further adsorption of Ni(II) and Cu(II) ions. Modification by **S** facilitates the adsorption of Ni(II) and Cu(II) ions.

Next, we will consider the second case involving the desorption of the Ca(II) and the addition of the Ni(II) and Cu(II) ions to the site where the Ca(II) ions were desorbed (Table 8).

Large positive values of Gibbs function changes indicate that this option for the adsorption of Ni(II) or Cu(II) ion adsorption with Ca(II) ion replacement is also unfavorable.

**Figure 13:**
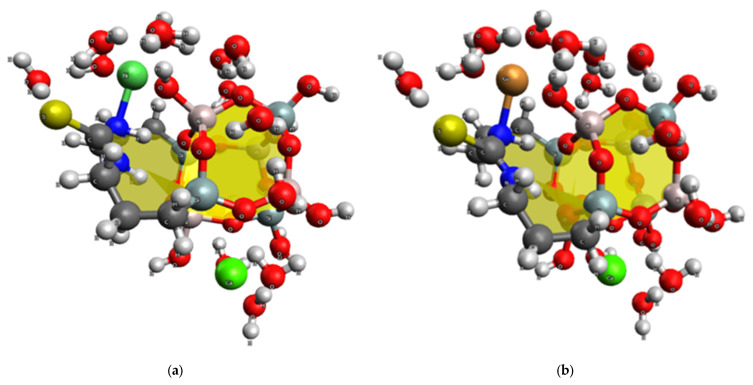
Cluster of **GS** with one Ca(II) ion and twelve H_2_O molecules and one Ni(II) ion (**a**) and one Cu(II) ion (**b**).

The third case shows the results of modeling the adsorption of Ni(II) and Cu(II) ions on the **GS** cluster alongside the desorption of Ca(II) ions. In this case, the Ni(II) and Cu(II) ions form a complex with the **S** residue via the nitrogen atoms of the thiocarbamide group and the oxygen atoms of the hydroxyl group of heulandite (Figure 13).

The thermodynamic functions for the addition of HMIs to the **GS** cluster, alongside parallel ion exchange between the Ca(II) ions and HMIs, were calculated (Table 9).

As can be seen, adsorption is thermodynamically favorable in this case. All of these ions are adsorbed more effectively than on heulandite **G** (Table 6).

**Figure 14:**
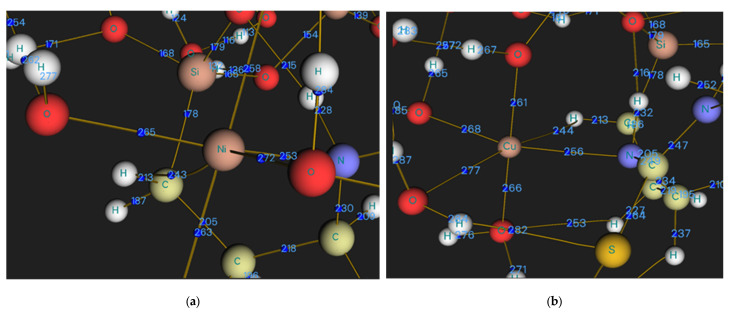
Bonding pathways and bond critical point (BCP) in the **GS**–HMI structure: (**a**) Ni bonding and (**b**) Cu bonding.

Figure 14 and Table 10 show the topological characteristics (bonding pathways and bond critical point (BCP) of the interactions between HMIs and the **GS** cluster).

As can be seen in Figure 14a,b, there are four types of interaction in the **GS** cluster system with HMIs. These are bonds between Ni(II) and Cu(II) ions and the nitrogen atom of the thicarbamide group **S** (BCP 253—Figure 14a; BCP 256—Figure 14b); specific bonds between Ni(II) and Cu(II) atoms with the hydrogen atom of the CH_2_ group (BCP 243—Figure 14a; BCP 244—Figure 14b); the bond between Ni(II) and Cu(II) ions and the hydroxyl group at Al (BCP 258—Figure 14a; BCP 261—Figure 14b), as well as the bonds between Ni(II) and Cu(II) ions and water molecules (BCP 263, 265, and 272—Figure 14a); BCP 266, 268, and 277—Figure 14b). There is no bond with sulfur.

Quantum chemical studies have shown that the adsorption of HMI on **GS** can occur via a mechanism of donor-acceptor interaction between heavy metal ions and donor nitrogen atoms of the modified sorbent.

Due to the low values of all parameters and the positive value of J, the interactions of metal ions with CH_2_ are weakly dispersive in nature. The Me-O and Me-N bonds are donor-acceptor, but in the case of Me-N, the proportion of covalency is greater. For the Cu(II) ion, the J values indicate stronger interaction stabilization than for the Ni(II) ion, due to the Cu(II) ion’s more pronounced polarizing ability.

## 3. Materials and Methods

The study was conducted on a natural zeolite from the Kholinskoye deposit, “Heulandite” (**G**), containing 25–30% by weight of the potassium feldspar KAlSi_3_O_8_ impurity. According to D. Breck’s classification [52], heulandite (D4) relates to group 7 of lamellar zeolites. The latter are ranked as microporous adsorbents characterized by micropore sizes ranging from 0.5 to 1.5 nm. CuSO_4_·5H_2_O (Merck), NiSO_4_·7H_2_O (Merck), (NH_4_)_2_SO_4_ (Merck), and hexane (Sigma) were brand reagents used without extra purification. N,N-bis(3-triethoxysilylpropyl)thiocarbamide (**S**) was prepared at A.E. Favorsky Irkutsk Institute of Chemistry. Cu^2+^ and Ni^2+^ standard solutions, having the concentration of a metal of 1 g/L, were obtained by dissolution of the sample in demineralized H_2_O. The adsorbed solutions were prepared using the dilution of the above standard solutions.

### 3.1. Synthesis of N,N-bis(3-Triethoxysilylpropyl)thiocarbamide (S) [34]

**Scheme 3:**
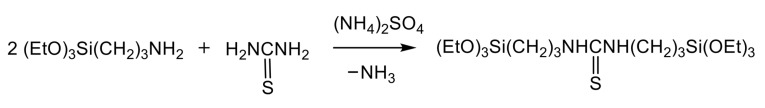
Synthesis of N,N-bis(3-triethoxysilylpropyl)thiocarbamide **(S)**.

A mixture of 3-aminopropyltriethoxysilane (0.2 mmol) and thiourea (0.1 mmol) in the presence of a catalyst (ammonium sulfate, 0.01 mmol) was stirred at 135°C until ammonia ceased to be released. The product was then washed repeatedly with ether and dried under vacuum (Scheme 3).

Yield 82%; yellowish oil; ^1^H NMR (400 MHz, CDCl_3_): δ 0.57 (m, 4H, SiCH_2_), 1.20 (t, 18H, CH_3_), 1.68 (m, 4H, -CH_2_-), 3.51 (m, 4H, NHCH_2_), 3.81 (q, 12H, OCH_2_). ^13^C NMR (100 MHz, CDCl_3_): δ 7.61 (SiCH_2_), 18.11 (CH_3_), 23.22 (CH_2_), 49.31 (NHCH_2_), 58.50 (OCH_2_). IR_max_ (cm^−1^): 3176 ν(NH), 1535 δ(NH) + ν(CN), 1230–1420 ν(C=S), 1085 ν(SiO), 960 ν(SiC).

### 3.2. Synthesis of Sorbent (GS)

Hexane (90 mL) was mixed with air-dried heulandite **G** (10 g, fraction 0.5–1.0 mm). Then, **S** (10 g) was added to the mixture in small portions over 10 min. The mixture was heated to 50 °C, with stirring for 1 h. The product was filtered off and sequentially washed with hexane and ethanol to remove excess **S**. The modified heulandite was then dried on air within 12 h and further in the oven at a temperature of 110 °C within 1 h. The adsorbent **GS** was employed to adsorb Cu^2+^ and Ni^2+^ from water solutions.

### 3.3. Determination of Morphology

Morphology of the adsorbents was evaluated using a Hitachi TM3000 scanning electron microscope (Hitachi High-Tech Corporation, Tokyo, Japan) having the magnification of up to 30,000X and the resolution of up to 25 nm. The samples were observed in 5 kV mode to allow defects in thin films on the surface under study to be detected. Using a backscattered electron detector enables volumetric samples to be observed with shadow and volumetric contrast. Surface elements were determined using energy-dispersive X-ray analysis (EDX) on a Quantax 70 detector (Bruker, Berlin, Germany). The EDX results are presented in the paper in mass %. Electron scanning of the samples was performed using a Quanta 200 FEI electron microscope (FEI Company, Eindhoven, The Netherlands).

### 3.4. Evaluation of the Textural Features of Heulandite and GS

Area of specific surface and the porosity of the materials were evaluated by the nitrogen adsorption/desorption Brunauer–Emmett–Teller (BET) method on SORBOMETER-M and TERMOSORB devices (Group of companies “GRANAT,” St. Petersburg, Russia), which are designed to study the textural features of the materials. These analyzers operate according to the nitrogen thermodesorption from the material surface in dynamic conditions. Following this approach, the helium–nitrogen steady flow of a material runs through the adsorber with the sample.

Prior to tests, the sample is degassed by heating in the stationary gas flow at a specified temperature to eliminate any gases that had been previously adsorbed onto its surface. The tests involved the following procedures: determining composition of the gas mixture; adsorbing gas adsorbate on the surface of the sample surface from a flow of gas mixture flow at a temperature of liquid nitrogen until equilibrium was reached between the gaseous adsorbate concentration in gas and adsorption phases; and desorbing gas adsorbate into a flow of gas mixture by heating the sample to a temperature at which the gaseous adsorbate was completely desorbed.

During the adsorption/desorption process, the volume part of gaseous adsorbate in a mixture is altered. This alteration is detected employing the heat conductivity coefficient of the detector. The heat conductivity detector’s output signal is a peak (converted into an electrical signal) of increased gas adsorbate concentration during its thermodesorption from the sample surface. The area of this peak is proportional to the volume of adsorbed gas desorbed from the sample.

### 3.5. PXRD

Powder X-ray diffraction analysis was carried out using a Bruker D8 ADVANCE instrument (Bruker Optik GmbH, Ettlingen, Germany) having a scintillation detector and a Gobel mirror (Cu-Kα radiation, 3–80° 2θ range) under the following conditions: 40 kV, 40 mA, 1 s per point, 2θ step 0.02°. The obtained data were analyzed by the DIFFRACplus software (version 6.0). The samples are identified by PDF-2 powder diffraction database (ICDD, 2007, www.icdd.com, accessed on 15 May 2025) and indexed by DIFFRAC.EVA (included in the DIFFRAC.SUITE v6.0 package, Bruker AXS).

**Scheme 4:**
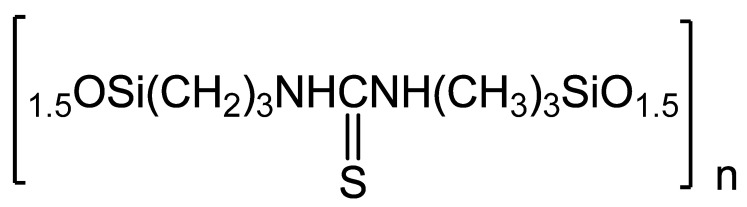
Siloxane SO.

The hydrolytic polycondensation solid product (H_2_O, pH 8–9), i.e., siloxane (**SO**) (Scheme 4), structurally related to organosilicon modifying agent **S,** was used as a comparison sample for XRD. The polymer comprises all of the structural motifs of **S** that are contained in the heulandite **GS**.

### 3.6. Adsorption Study

The experiments used a pre-sieved fraction of heulandite **G** and sorbent **GS** (0.5–1.0 mm). Ion adsorption was studied in aqueous solutions prepared using CuSO_4_ • 5H_2_O, NiSO_4_ • 7H_2_O, and deionized water. The initial concentration of metal ions in the solutions was based on the actual composition of wastewater from electroplating production. The heavy metal ion concentration spanned 5–100 mg/l. In each solution, the metal ion equilibrium concentration was determined by FAAS (inductively coupled plasma atomic emission spectroscopy method on a Varian Spectra Plus atomic absorption spectrophotometer (Varian, Inc., Santa Clara, CA, USA)). Standard single-component solutions (CuSO_4_ and NiSO_4_) with a copper and nickel content of 1000 mg/L were used to prepare calibration solutions. Working calibration solutions (five concentrations) covering the linear range of the device for the corresponding element lamp were prepared from the primary solution by sequential dilution. For Cu and Ni, range was 0.1, 0.5, 1.0, 2.5, and 5.0 mg/L. A nitric acid solution with a molar concentration of 0.3 mol/L was used as the blank solution. All measurements were performed at least three times. The linearity of the calibration curve was confirmed by a coefficient of determination R^2^ = 0.999. The intermediate repeatability and reproducibility index was ± 6%.

Quality control of metal determination was carried out using the addition method. Selected samples were analyzed using the standard addition method. The results obtained were in good agreement (discrepancy < 8%), with the results obtained from the calibration curve in a pure environment, confirming the absence of significant matrix effects.

Adsorption characteristics of heulandite **G** and sorbent **GS** relative to Cu^2+^ and Ni^2+^ were examined in static conditions. Isotherms of the adsorption were constructed using the constant weight method (1 g of heulandite) and changeable metal ion concentrations. Values of the adsorption (*A*, mg/g) were determined using the following formula:

*A* = (*C*_0_ − *C*eq)*V*/*m*,

where C_0_ and Ceq are the starting and equilibrium concentrations of Me^+^ in a solution (mg/l); *m* is the adsorbent mass (g); *V* is the solution volume (l); and m is the sorbent mass. The solution volume was 0.05 l. The sorbent mass was 0.5 g. To analyze the isotherms, the runs on elimination of Cu^2+^ and Ni^2+^ were performed two times. In further calculation, mean value was employed.

The model solutions acidity was measured on a pH-340 potentiometer (Russian Federation) following the typical procedure. Temperature was kept at 298 K by a UTU-4 thermostat. A magnetic stirrer (500 rpm) was used for stirring the solutions. The conditions of the stirring were intact during all the procedures: Ni(II) ion sorption—initial pH = 5.9; as a result of sorption, acidification occurs to pH = 5.4. Specific electrical conductivity: initial 147.1 μS/cm and final 155.4 μS/cm. Cu(II) ion sorption—initial pH = 5.05; as a result of sorption, acidification occurs to pH = 4.8. Specific electrical conductivity: initial 97.4 μS/cm and final 115.8 μS/cm.

Natural zeolite undergoes alkalization due to the sorption of Cu(II) and Ni(II) ions (Table 11).

It is known that the adsorption of heavy metal ions (HMI), including Ni(II) ions, is most effective at a pH close to the hydration values of the ions under study [53]. Thus, a pH of 6 corresponds to the start of Ni(OH)_2_ precipitation. The maximum release of Ni(OH)_2_ is observed at pH = 9–10. Therefore, it is preferable to carry out the adsorption of Ni(II) ions at pH = 6 or below, which is consistent with the literature data [54].

### 3.7. Mathematical Modeling of Experimental Adsorption Data

The experimental data on the adsorption of Cu(II) and Ni(II) ions by the **GS** sample were processed using the Statgraphics Plus software. The type of regression was selected based on the maximum value of the coefficient of determination *R*_2_, % [55]. The R_2_ value shows what percentage of the experimental data is described by the obtained regression equation. The closeness of the relationship between the dependent and independent variables is described by the adjusted coefficient of determination *R*c_2_, and the accuracy of the model is described by the root mean square σ and absolute Δ errors.

### 3.8. Computer Modeling (See Supplementary Materials)

Computer modeling was performed using version 6.0.1 of the Orca software package [56,57,58,59]. Density functional theory was chosen as the modeling method, using the nonlocal density functional PBE [60,61]. To account for fine dispersion interactions in the considered atomic–molecular systems, the D4 dispersion correction was also applied [62,63,64,65]. The def2-SVP basis set [66] was chosen for light atoms (H, C, N, O, Al, and Si). For metal atoms (Ca, Ni, and Cu), more complete and accurate def2-TZVPP basis set was chosen [67]. For hardware acceleration of the calculation of Coulomb integrals, an additional basis set def2/J [63], the RI-J method [68], and the SHARK integral solution algorithm [69] were employed. Each atomic–molecular system under consideration was placed in a dielectric cavity with specified values of dielectric permittivity (ε = 80.15) and refractive index (n = 1.333) to account for the solvent effect (water). The solvent was modeled using the continuous CPCM (Conductor-like Polarizable Continuum Model) method [70]. The cavity surface type is Gaussian VdW with surface charge blurring [39,71]. After optimizing the atomic–molecular systems, the oscillation frequencies were calculated analytically. This was followed by an evaluation of the thermodynamic functions (enthalpy H, entropy S, and Gibbs function G). All thermodynamic functions were calculated under standard conditions (T = 298.15 K and p = 1 atm). The absence of imaginary vibration frequencies indicates a true minimum on the potential energy surface.

To model the effects of hydration completely, explicit H_2_O water molecules were added to all the structures that were modelled, in addition to simple CPCM accounting. The water molecules were added to the system using the Docker tool from the Orca 6.0.1 package. Each water molecule was embedded in the cluster through docking by taking into account the principle of minimum energy and by statistically enumerating different possible positions.

The thermodynamics of adsorption were studied by evaluating the difference in the corresponding thermodynamic potentials based on the quantum dimension principle and the consequences of Hess’s law.

The structure of heulandite was modeled as an aluminosilicate cluster containing two calcium ions Ca(II). The **G** cluster model from [72] was chosen for its balance of accuracy and calculation time.

To evaluate the types of interactions that are present in the aluminosilicate cluster-modifying molecule–adsorbate system, Beider’s Quantum Theory of Atoms in Molecules was applied [72]. The corresponding topological analysis of electron density was performed using the version of 3.8 Multiwfn software package [73].

## 4. Conclusions

1. A new adsorbent (**GS**) was obtained by modifying the zeolite “heulandite” (**G**) with N,N′-bis(3-triethoxysilylpropyl)thiocarbamide (**S**). The composition, structure, and surface morphology of the adsorbent **GS** were confirmed using elemental analysis, IR-, NMR-spectroscopy, XRD, SEM, EDX spectra, elemental mapping, and nitrogen adsorption/desorption by the BET method. The potential of the obtained materials for the application as sorbents for the removal of Cu(II) and Ni(II) ions from concentrated solutions has been revealed. The nature of HMIs adsorption was investigated using the Langmuir, Freundlich, and Dubinin–Radushkevich models.
2. The adsorption capacity of Cu(II) and Ni(II) ions by **GS** sorbents was found to be 1.7 and 2.1 times higher than that of heulandite **G**, amounting to 0.128 mmol/g (8.1 mg/g) and 0.214 mmol/g (12.6 mg/g), respectively. It was established that the free energy of adsorption E during the adsorption of Cu(II) and Ni(II) ions was 12.5 and 16.2 kJ/mol, respectively. Calculations of changes in Gibbs energy based on quantum chemical modeling results ($\Delta G_{298}^{0}$ = −38.5 kJ/mol for Ni and $\Delta G_{298}^{0}$ = −56.5 kJ/mol for Cu) confirmed that adsorption of heavy metal ions onto the GS sample occurs through the formation of metal ion coordination complexes with the sorbent’s functional groups (chemosorption).
3. It has been found that in heulandite **G**, Ca(II) ions are strongly bound to oxygen atoms in the aluminosilicate framework through electrostatic, closed-shell interactions (ionic bonds). Modification of the heulandite structure with N,N′-bis(3-triethoxysilylpropyl)thiocarbamide **S** weakens the bond between the oxygen and calcium atoms. This increases the desorption capacity of calcium ions, promoting the subsequent adsorption of nickel and copper ions. Changes in Gibbs energy calculated based on the results of quantum chemical modeling ($\Delta G_{298}^{0}$ = −18.55 kJ/mol for nickel and $\Delta G_{298}^{0}$ = −2.08 kJ/mol for copper) confirm that nickel adsorption is an ion-exchange process, while the adsorption Cu(II) ion is a physical process involving the micropores of the heulandite **G** sorbent.
4. The adsorption of Ni(II) and Cu(II) ions on **GS** occurs primarily through the donor-acceptor interactions between these ions and the nitrogen atom, as well as the OH group at the heulandite aluminum atom. There are no bonds with sulfur; however, electrostatic interactions occur between the metal ion and the electron-rich group of aluminosilicate [AlO_3_(OH)]^−^.
5. The presence of strong polarization effects and donor-acceptor interactions has been proven to cause good contact between Ni(II) and Cu(II) ions, as well as nitrogen and sulfur atoms in structures modified with N′-bis(3-triethoxysilylpropyl)thiocarbamide. Interaction with oxygen atoms of heulandite is not always favorable. Based on the topological analysis of electron density values, it can be concluded that calcium ions prefer to interact with oxygen atoms, whereas nickel and copper ions prefer to interact with nitrogen atoms.

## Figures and Tables

**Table 1:** Molecular formulas and textural characteristics of heulandite and clinoptilolite.

Zeolite	Idealized Molecular Formula	Typical Si/Al Ratio	Fraction Diameter, mm	Specific Surface Area, cm^2^/g	Average Pore Size, nm
Clinoptilolite	Na_6_[(AlO_2_)_6_(SiO_2_)_30_]·24H_2_O	6.5	0.5–1	34	1.7
Heulandite	Ca_4_[(A1O_2_)_8_(SiO_2_)_28_]·24H_2_O	3.5	0.5–1	32	1.8

**Table 2:** Parameters of the Langmuir adsorption model.

	*A*_∞_, mmol/g	*A*_∞_, mg/g	*K·*10^−3^	Linear Form of the Langmuir Equation	*R_L_*	*R* ^2^
Cu(II) ions						
**G**	0.087	5.5	33.80	1/*A* = 0.342·1/*C*_eq_ + 11.552	0.04	0.999
**GS**	0.468	29.7	14.16	1/*A* = 0.151·1/*C*_eq_ + 2.138	0.09	0.996
Ni(II) ions						
**G**	0.150	8.8	14.07	1/*A* = 0.474·1/*C*_eq_ + 6.670	0.06	0.992
**GS**	0.781	46.1	8.06	1/*A* = 0.159·1/*C*_eq_ + 1.281	0.10	0.947

**Table 3:** Parameters of the Freundlich adsorption model.

Sorbents	Linear Form of the Freundlich Equation	*K_f_*	*n*	*R* ^2^
Cu(II) ions				
**G**	log *A* = −0.741 + 0.455·log *C*_eq_	0.182	2.20	0.969
**GS**	log *A* = −0.513 + 1.379·log *C*_eq_	0.307	0.73	0.966
Ni(II) ions				
**G**	log *A* = −0.581 + 0.493·log *C*_eq_	0.262	2.03	0.962
**GS**	log *A* = −0.446 + 0.520·log *C*_eq_	0.358	1.92	0.954

**Table 4:** Parameters of the Dubinin–Radushkevich adsorption model.

Sorbents	Linear Form of the Dubinin–Radushkevich Equation	*A_m_*, mmol/g	*k*, mol^2^/kJ^2^	*E*, kJ/mol	*R* ^2^
Cu(II) ions					
**G**	−ln *A* = −2.295 + 0.008 ɛ^2^	0.101	0.008	7.9	0.998
**GS**	−ln *A* = −2.639 + 0.003 ɛ^2^	0.071	0.003	12.5	0.934
Ni(II) ions					
**G**	−ln *A* = −1.874 + 0.007 ɛ^2^	0.154	0.007	8.5	0.987
**GS**	−ln *A* = −1.608 + 0.0023 ɛ^2^	0.200	0.002	14.7	0.928

**Table 5:** Thermodynamic functions for ion addition to cluster **G**.

Thermodynamic Functions	HMI	
	Ni(II) Ions	Cu(II) Ions
$\Delta H_{298}^{0}$, kJ/mol	−19.42	+0.12
$\Delta S_{298}^{0}$, J/mol·K	−2.94	+7.38
$\Delta G_{298}^{0}$, kJ/mol	−18.55	−2.08

**Table 6:** Thermodynamic functions for the attachment of ions to the **GS** cluster without the desorption of Ca(II) ions.

Thermodynamic Functions	HMI	
	Ni(II) Ions	Cu(II) Ions
$\Delta H_{298}^{0}$, kJ/mol	+63.17	+80.73
$\Delta S_{298}^{0}$, J/mol·K	+2.00	+32.74
$\Delta G_{298}^{0}$, kJ/mol	+60.01	+70.97

**Table 7:** Thermodynamic functions for the removal of the first calcium ion from the **GS** cluster.

Thermodynamic Functions	Values of the First Ion of Ca(II)
$\Delta H_{298}^{0}$, kJ/mol	−16.58
$\Delta S_{298}^{0}$, J/mol·K	+49.59
$\Delta G_{298}^{0}$, kJ/mol	−31,36

**Table 8:** Thermodynamic functions for the addition of HMIs to the **GS** cluster.

Thermodynamic Functions	HMI	
	Ni(II) Ions	Cu(II) Ions
$\Delta H_{298}^{0}$, kJ/mol	+54.66	+68.34
$\Delta S_{298}^{0}$, J/mol·K	+48.36	+66.06
$\Delta G_{298}^{0}$, kJ/mol	+40.24	+48.94

**Table 9:** Thermodynamic functions for the addition of HMIs to the **GS** cluster.

Thermodynamic Functions	HMI	
	Ni(II) Ions	Cu(II) Ions
$\Delta H_{298}^{0}$, kJ/mol	−58.58	−73.26
$\Delta S_{298}^{0}$, J/mol·K	−67.38	−56.06
$\Delta G_{298}^{0}$, kJ/mol	−38.5	−56.54

**Table 10:** Topological characteristics of **GS**-metal ion complexes.

Structure		GS + Ni(II)–Ca(II)	GS + Cu(II)–Ca(II)
Ρ	Me—H_2_O	0.074	0.081
	Me—O_Al_	0.079	0.088
	Me—N	0.062	0.068
	Me—CH_2_	0.007	0.007
Δρ	Me—H_2_O	0.397	0.407
	Me—O_Al_	0.430	0.444
	Me—N	0.237	0.254
	Me—CH_2_	0.022	0.020
*J*	Me—H_2_O	−0.106	−0.156
	Me—O_Al_	−0.124	−0.176
	Me—N	−0.133	−0.180
	Me—CH_2_	+0.043	+0.042
Atomic charge	Me	+1.131	+1.076
	S	−0.011	+0.011
	N	−1.132	−1.118
	O_Al_	−1.421	−1.400

**Table 11:** pH values of hydrate formation.

Hydroxide	pH of the Beginning of Hydrate Formation	pH Max of Hydroxide Release	pH of the Beginning of Hydroxide Dissolution
Cu(OH)_2_	5.3	8.0–9.5	>9
Ni(OH)_2_	6.0	9.25–10	-

## Data Availability

The original contributions presented in this study are included in the article/Supplementary Material. Further inquiries can be directed to the corresponding author.

## Supplementary Materials

The following supporting information can be downloaded at: https://www.mdpi.com/article/10.3390/molecules30244811/s1.
