# Supplementary material for: Zeolite Heulandite Modified with N,N′-bis(3-Triethoxysilylpropyl)thiourea—Adsorption of Ni(II) and Cu(II) Ions: A Quantum Chemical Insight into the Mechanism
Source: Molecules. 2025 Dec 17;30(24):4811. doi: 10.3390/molecules30244811 (PMC12736353; doi:10.3390/molecules30244811)
Supplement: Supplementary file 1 [file molecules-30-04811-s001.zip › molecules-4011559-supplementary.pdf]

Zeolite Heulandite Modified with N,N′-bis(3-Triethoxysilylpropyl)thiourea—Adsorption of Ni(II) and Cu(II) Ions: A Quantum Chemical Insight into the Mechanism

Elena G. Filatova, Arailym M. Nalibayeva, Oksana V. Lebedeva, Sergey A. Beznosyuk, Andrey V. Ryabykh, Elizaveta N. Oborina, Yerlan N. Abdikalykov, Mirgul Zh. Turmukhanova, Igor B. Rozentsveig, Sergey N. Adamovich *

Quantum chemical study of the adsorption mechanism of GS by heavy metal ions (Additional explanations):

1) The PBE functional provides an effective balance between computational cost and the accuracy with which it describes geometry, bond energies and non-bonding interactions in systems containing similar atoms (O, Al, Si, Ca, Ni, Cu). When used alongside the D4 dispersion correction, the PBE functional can accurately describe covalent and ionic interactions within metal frameworks and complexes, as well as weak intermolecular bonds. This includes the polarisation and coordination of water molecules with ions and the framework.

2) The convergence parameters for SCF self-consistency and geometry optimization procedures are defined within ORCA using the NormalSCF and NormalOPT commands, respectively.

3) In order to adequately describe the specific ion-dipole and ion-ion interactions that are essential for understanding the ion exchange mechanism, we chose a hybrid approach to account for the presence of the aqueous environment: continuous CPCM and the inclusion of 12 explicit water molecules. To simulate free ions (Ca2+, Ni2+, Cu2+), at least 6 water molecules are required to enter the first coordination sphere of the ion. The remaining six molecules form the second coordination sphere. A series of preliminary simulations were performed with different numbers of water molecules at the ions and the aluminosilicate cluster. Based on the balance between time and calculation accuracy, 12 water molecules were selected for use. Consequently, all participants in the adsorption process had the same number of explicit water molecules.
The position of explicit water molecules was optimized using the Solvator tool in the ORCA package. This tool systematically searches through possible positions to find the configuration with the lowest energy, ensuring a realistic model of the hydration shell is produced.
To confirm that the true minimum had been found on the potential energy surface for each optimised structure, the vibrational frequencies were calculated using the harmonic approximation (AnFreq). The absence of imaginary frequencies was established in all cases. Based on the frequency analysis, corrections to the energy and thermodynamic functions (enthalpy H, entropy S, Gibbs function G) were calculated under standard conditions.
